# Cinnamic Acid Inhibited Growth of Faba Bean and Promoted the Incidence of Fusarium Wilt

**DOI:** 10.3390/plants7040084

**Published:** 2018-10-11

**Authors:** Qian Zhao, Ling Chen, Kun Dong, Yan Dong, Jingxiu Xiao

**Affiliations:** College of Resources and Environment, Yunnan Agricultural University, Kunming 650201, China; z07070419@163.com (Q.Z.); qzyauedu@163.com (L.C.); 2000028@ynau.edu.cn (K.D.); 2005023@ynau.edu.cn (J.X.)

**Keywords:** allelopathy, faba bean, *Fusarium oxysporum* f. *fabae* (FOF), pathogenicity, phenolic acid

## Abstract

To ascertain the role and mechanism of cinnamic acid in the process of soil-borne Fusarium wilt infection with fava bean, we studied the effect of cinnamic acid on the faba bean and *Fusarium oxysporum* f. *fabae* (FOF). Our results showed that cinnamic acid treatment affected the physiological resistance of faba bean to FOF after inoculation with the pathogen and enhanced the pathogenicity of the pathogen, which may have led to aggravation of infection by the pathogen and increases in the incidence rates of Fusarium wilt and disease.

## 1. Introduction

The faba bean (*Vicia faba* L.) is an important leguminous crop for multitudes worldwide. It is widely used as food, fodder, and green manure, and has high adaptability and high nitrogen fixation. In recent years, faba bean production has been under continuous development to improve its yield under various planting and environmental conditions. The faba bean tends to experience obstacles to replantation after continuous cropping, especially soil-borne diseases and low yield [1].

Fusarium wilt is among the main soil-borne diseases threatening the production of faba bean [2], as well as many other crops, including cucumber (*Cucumis sativus* L.), watermelon (*Citrullus lanatus* (Thunb.) Matsum. et Naka), and strawberry (*Fragaria ananassa* Duch.) [3,4,5]. This disease is principally caused by *Fusarium oxysporum* [6], which infects plants by interfering with signal recognition in host plants and destroying plant defence systems.

Many researchers have found that allelopathy and autotoxicity are closely related to problems in continuous cropping, with connections made to numerous allelochemicals. Phenolic acid is the most widely studied and active of these allelochemicals [7]. Researchers have isolated more than 10 types of phenolic acid from root exudates and decomposed residue of crops, such as watermelon, eggplant (*Solanum melongena* L.), lily (*Lilium brownii* var. *viridulum* Baker), and radix rehmanniae (*Rehmannia glutinosa* (Gaetn.) Libosch. ex Fisch. et Mey.) [8,9,10,11]. Among these, cinnamic acid is one of the most important phenolic acids [12]. Asaduzzaman and Asao [13] found that the addition of activated charcoal to hydroponic culture improved the growth of faba bean and the detection of several phenolic acids, including cinnamic acid, and that its self-toxic substances had an important inhibitory effect on plant growth.

Studies have shown that phenolic acids have a stimulating effect (positive or negative) on soil-borne pathogens. One study showed that *p*-hydroxybenzoic acid, vanillic acid, and ferulic acid, which are root exudates of cucumber, stimulated mycelial growth in *F. oxysporum* [14], and the experimental results of Wang et al. [15] showed that cinnamic acid inhibited the spore germination of *Verticillium dahliae* in eggplants. The stimulating effect of phenolic acids on pathogenic fungi has been linked to its concentrations in several studies. For example, in American ginseng (*Panax quinquefolius* L.), phenolic acid promoted *Fusarium solani* growth at concentrations of 0.5 and 0.75 mmol·L^−1^, whereas 1 mmol·L^−1^ inhibited growth [16].

In our early research, we detected seven phenolic acids in rhizosphere soil that continuous cropped faba bean, with cinnamic acid being the most abundant component [17]. However, the role of cinnamic acid, which constitutes the highest proportion of phenolic acid from faba bean root exudates, has not been reported in the process of soil-borne fusarium wilt infection, nor its mechanism.

Therefore, the objective of the current study was to examine the effects of cinnamic acid on the growth of faba bean seedlings. The effects of cinnamic acid on Fusarium wilt, antioxidant enzymes, and disease-related proteins in faba bean were studied using cinnamic acid treatments and inoculation of the pathogen. The mechanism of Fusarium wilt promotion by cinnamic acid in faba bean was explored from the perspective of the host and pathogen, to evaluate the effect of cinnamic acid on *Fusarium oxysporum* f. *fabae* (FOF) pathogenicity.

## 2. Results

### 2.1. Effect of Cinnamic Acid on Plant Growth

Growth parameters of faba bean all significantly decreased following cinnamic acid treatment, except for main root length and root–shoot ratio (Table 1). Cinnamic acid treatment significantly reduced the number of leaves per plant, by 24.64%, 40.01%, and 45.41% at cinnamic acid concentrations of 50, 100, and 200 mg·L^−1^, respectively, compared with the control. Maximum leaf length declined by 20.28%, 24.15%, and 47.25%; maximum leaf width by 23.94%, 30.57%, and 46.04%, height by 15.29%, 30.33%, and 41.80%; shoot dry weight by 25.56%, 58.74%, and 68.16%; and root dry weight by 27.28%, 62.96%, and 74.07%, respectively (Table 1).

### 2.2. Effect of Cinnamic Acid on Fusarium Wilt Incidence and Disease Indices

**Figure 1:**
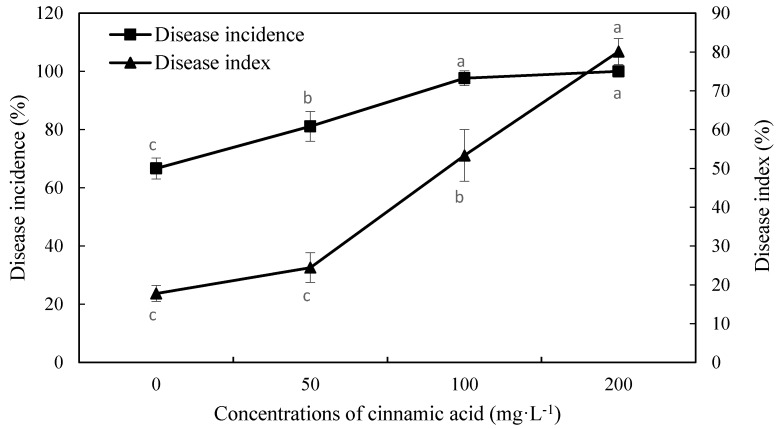
Effects of cinnamic acid on the incidence and index of faba bean Fusarium wilt. Data are average values, with bars representing standard errors of three replicates. Different letters for each index indicate significant differences at *p* < 0.05, according to Duncan’s test. There are no significant differences among values labeled “ab”, “a”, and “b”.

The incidence of Fusarium wilt increased significantly under cinnamic acid stress at concentrations of 50, 100, and 200 mg·L^−1^, and also increased as cinnamic acid content increased (Figure 1). Compared with the control, Fusarium wilt incidence increased by 21.67%, 46.5%, and 50% with treatments of 50, 100, and 200 mg·L^−1^, respectively, and the disease index significantly increased by 37.5%, 200%, and 350.62% at treatment concentrations of 50, 100, and 200 mg·L^−1^ (Figure 1). In general, cinnamic acid led to increased Fusarium wilt incidence, and stimulated the conditions of the disease and its effects in a concentration-dependent manner.

### 2.3. Effect of Cinnamic Acid on Seedling Root Physiological Activity 

Peroxidase (POD) and catalase (CAT) activities exhibited a significant negative relationship with cinnamic acid treatment concentration, decreasing in an almost linear manner as treatment concentration increased (Table 2). POD activity was inhibited by 17.14%, 30%, and 48.57% at cinnamic acid concentrations of 50, 100, and 200 mg·L^−1^, respectively, and CAT activity was reduced by 15.63%, 44.77%, and 62.97% (Table 2).

Malondialdehyde (MDA) content increased in faba bean seedling roots under cinnamic acid stress at almost all concentrations (Table 2) in a concentration-dependent manner.

### 2.4. Effect of Cinnamic Acid on Pathogenesis-Related Faba Bean Seedling Root Proteins

**Figure 2:**
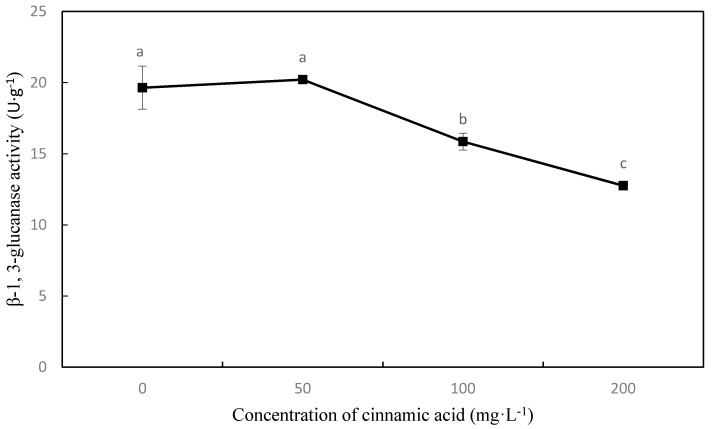
Effects of cinnamic acid on β-1, 3-glucanase activity in faba bean roots after being inoculated with FOF. Data are average values, with bars representing standard errors of three replicates. Different letters for each index indicate significant differences at *p* < 0.05, according to Duncan’s test. There are no significant differences among values labeled “ab”, “a”, and “b”’.

**Figure 3:**
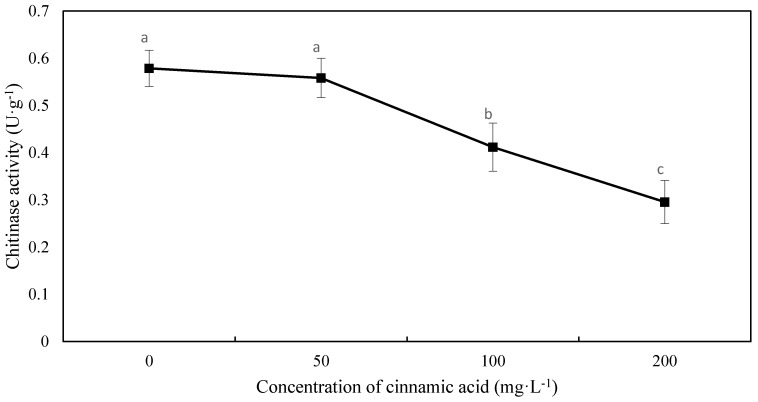
Effects of cinnamic acid on chitinase activity in faba bean roots after being inoculated with FOF. Data are average values, with bars representing standard errors of three replicates. Different letters for each index indicate significant differences at *p* < 0.05, according to Duncan’s test. There are no significant differences among values labeled “ab”, “a”, and “b”’.

Seedling root β-1,3-glucanase activity showed a decreasing trend as cinnamic acid concentration increased, except at 50 mg·L^−1^ where it showed a slight stimulating effect. With 100 and 200 mg·L^−1^ treatments of cinnamic acid, β-1,3-glucanase activity decreased by 19.31% and 35.07%, respectively, compared with the control (Figure 2). The chitinase activity trend was similar to that of β-1,3-glucanase activity, and compared with the control, had significant decreases at 100 and 200 mg·L^−1^ by 28.84% and 48.94%, respectively (Figure 3).

### 2.5. Effect of Cinnamic Acid on FOF Mycotoxin Production

**Figure 4:**
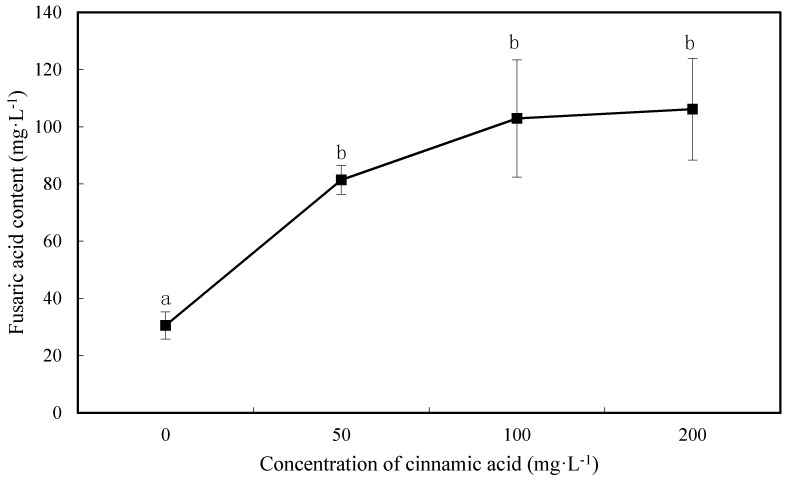
Effects of cinnamic acids at various concentrations on mycotoxin production of FOF. Data are average values, with bars representing standard errors of three replicates. Different letters for each index indicate significant differences at *p* < 0.05, according to Duncan’s test. Significant differences are labelled “a”, “ab”, and “b”.

FOF mycotoxin yield (FA) increased as cinnamic acid concentration increased, with a significant increase at 50 mg·L^−1^ (Figure 4). FA yield increased by 166.32%, 236.58%, and 247.12%, respectively, at concentrations of 50, 100, and 200 mg·L^−1^, compared with the control (Figure 4).

### 2.6. Effect of Cinnamic Acid on the Activities of Hydrolytic Enzymes Related to Pathogenesis

**Figure 5:**
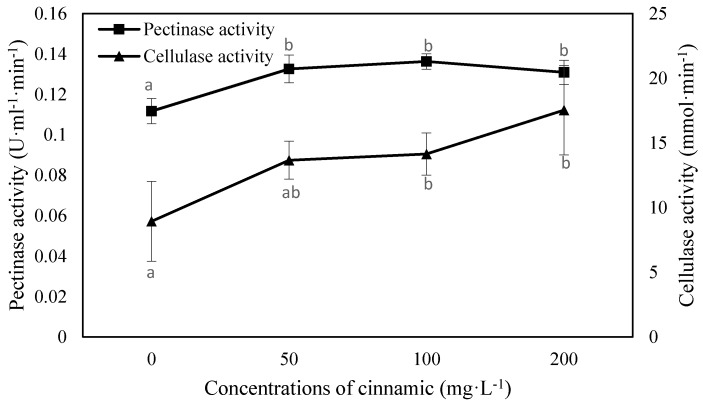
Effects of cinnamic acid at various concentrations on pectinase and cellulase activity of FOF. Data are average values, with bars representing standard errors of three replicates. Different letters for each index indicate significant differences at *p* < 0.05, according to Duncan’s test. Significant differences are labelled “a”, “ab”, and “b”.

A slight increase in pectinase activity was observed under cinnamic acid stress, with increases of 18.67%, 21.96%, and 17.15%, respectively (Figure 5). Cellulase activity increased, then remained stable at 50–100 mg·L^−1^ cinnamic acid, and then accelerated as the treatment concentration increased. With increasing concentrations of cinnamic acid, cellulase activity increased by 52.77%, 58.19%, and 96.08%, at 50, 100, and 200 mg·L^−1^, respectively (Figure 5).

## 3. Discussion

The role of phenolic acids has become a research hot spot in the field of autotoxicity. A study by Hu et al. [14] reported that exogenous application of *p*-hydroxybenzoic acid, vanillic acid, and ferulic acid slightly reduced plant height, root, stem, and leaf dry weight in cucumber seedlings, and induced seedling stem diameter narrowing and root length shortening. Other studies have shown that phenolic acids can have a hormetic effect on the seed germination or growth of plants [18,19], which is consistent with our results. In the current study, faba bean seedling growth was significantly inhibited under cinnamic acid and FOF stress, demonstrated by reduction of the number of leaves per plant, plant height, shoot dry weight, and root dry weight. This inhibitory effect increased as treatment concentration increased, and reached a significant level at 50 mg·L^−1^ cinnamic acid (Table 1). This result indicates that cinnamic acid plays a role in inhibiting the growth of faba bean seedlings infected with FOF in a concentration-dependent manner.

Self-toxic substances also have an effect on the occurrence of soil-borne diseases, which can inhibit crop growth and reduce economic efficiency. In a study of continuous cropping in strawberries, ferulic and coumaric acids were detected. Continuous cropping soil was found to significantly promote strawberry anthracnose crown rot [20]; Qi et al. [21] reported that strawberry root tissues were significantly damaged under stress by p-hydroxybenzoic acid and *Fusarium oxysporum*, promoting the occurrence of strawberry blight. In the current study, the incidence of *F. oxysporum* in faba bean at the three treatment concentrations reached 81.11, 97.67, and 100%, respectively, being 21.67%, 46.5%, and 50% higher than that of the control. The disease index also increased significantly, by 200 and 350.62% at 100 and 200 mg·L^−1^, respectively. These results are similar to those of Wang [22] who detected a significant increase in the incidence of fusarium wilt on watermelon seedlings; the disease index and seedling death rate increased as benzoic and cinnamic acid concentrations increased.

As the length of continuous cropping increases, autotoxic compounds accumulate in the soil. When these toxic substances reach a critical amount, the plant’s physiological resistance will change, inducing a decline in resistance and tissue damage. Superoxide dismutase (SOD), POD, and CAT activities have been shown to increase in chili (*Capsicum annuum* L.) leaves planted in continuous cropping soil, and content of MDA and osmotic substances increased concurrently [23]. However, a study on *Solanum melongena* indicated that after years of continuous cropping, a large number of allelochemicals, including vanillin, had accumulated in the soil, leading to a decrease in root activity and chlorophyll content in tomato leaves at a 4.0 mmol·L^−1^ concentration of vanillin; this was because the content of MDA and free proline (Pro) increased, and the relative permeability of the cell membrane increased [24]. Studies on strawberry, apple (*Malus pumila* Mill.), cucumber, and American ginseng showed that phenolic acid treatment reduced protective enzyme activities and increased cell membrane permeability [25,26,27,28]. These results are consistent with those of the current study, which showed that cinnamic acid treatment significantly reduced POD and CAT activity in faba bean roots and significantly increased MDA content, indicating that cinnamic acid and FOF stress reduced the activities of protective enzymes and stimulated lipid peroxidation in roots, weakening the physiological resistance of faba bean and promoting pathogen infection. These combined factors increased the rate of occurrence of Fusarium wilt in this species.

When host plants are infected by pathogenic bacteria, their defence response is initiated and the expression of disease-related proteins is induced, producing important hydrolases (e.g., chitinase and β-1,3-glucanase) that hydrolyse the fungal cell wall, inhibiting fungal growth [29]. In soybean (*Glycine max* (Linn.) Merr.), resistance to *Phytophthora sojae* was positively correlated with β-1,3-glucanase and chitinase activity [30]. Other studies have shown that self-toxic substances are promoted under peroxidation stress and have an impact on the plant defence system. Appropriate concentrations of p-hydroxybenzoic acid have been shown to enhance the induction of chitinase and β-1,3-glucanase activity, improving the resistance of Chinese cabbage (*Brassica campestris* L. ssp. *chinensis* var. *utilis* Tsen et Lee) to anthracnose [31]. Other studies have demonstrated that phenolic acids have a negative effect on chitinase and β-1,3-glucanase activity. Exogenous application of ferulic acid and inoculation with *F. oxysporum* have been shown to significantly inhibit chitinase activity in watermelon roots in a concentration-dependent manner, and has the greatest effect with the application of 100 μmol·L^−1^ ferulic acid, leading to reductions of 62.5% in chitinase activity and 32.0–37.0% in β-1,3-glucanase activity in watermelon leaves [32]. In the current study, chitinase and β-1,3-glucanase activities were significantly lower than those of the control when cinnamic acid was applied at a concentration exceeding 50 mg·L^−1^, indicating that cinnamic acid destroyed the defence system against FOF, thereby decreasing faba bean resistance against pathogenic bacteria and resulting in serious wilt disease.

Several allelochemicals can cause problems in continuous cropping. Of these, phenolic acids are the most studied, as well as the most active [33,34]; they have been shown to act as allelochemicals in root exudates [35,36,37], inhibit crop growth, and stimulate pathogens. The amount of *F. oxysporum* in the matrix increased by 76.7% and 104.6% when strawberry was treated with 0.2 and 0.4 mmol·L^−1^ p-hydroxybenzoic acid, respectively [38]. Ferulic acid, a watermelon root exudate, significantly promoted *F. oxysporum* f. Sp. spore germination and sporulation, with promotion rates of 28.6–114.3% and 17.7–54.8%, respectively, at concentrations of 40–160 mg·L^−1^ [37]. During the infection process, the hydrolysis of enzymes (pectinase and cellulase) and pathogenic toxins is an important virulence factor that promotes the development of many plant fungal diseases and degrades cell wall tissues. Cell wall hydrolases promote host invasion by pathogen mycelia; the pathogen then secretes toxins, interferes with host metabolic functions, and destroys the plasma membrane, mitochondria, and chloroplasts [39,40,41]. In turn, membrane permeability is changed, affecting photosynthesis and respiration [42,43], inhibiting DNA, RNA, and other substances, as well as protein biosynthesis [44,45]. Studies have shown that the pathogenicity is closely related to enzyme and toxin production [46,47]. The results of the current study show that exogenous application of cinnamic acid promoted the production of *F. oxysporum* and hydrolytic enzymes at almost all concentrations. Thus, cinnamic acid promotes FOF infectivity and pathogenicity during infection.

However, these approaches, such as the application of bio-organic fertilizers, intercropping, mixed cropping, and rotation, can alleviate the effect of replanting obstacles and inhibit the occurrence of soil-borne diseases. Studies have shown that intercropping systems enrich the soil’s microbial community structure, accelerate degradation of autotoxins such as cinnamic acid, reduce the self-toxic effect, and improve the physiological resistance of plants by resisting infection by pathogens and increasing crop yield [48,49,50].

## 4. Materials and Methods

### 4.1. Test Materials

The faba bean variety (*Vicia faba* L.), 89–147, used in this study was purchased from the Yunnan Academy of Agricultural Sciences (Yunnan Sheng, China). Analytical-grade cinnamic acid was purchased from China Pharmaceutical Group Shanghai Medical Instrument Co., Ltd. (Shanghai, China).

FOF was isolated from monocropped faba bean fields by the Plant–Microbe Laboratory at Yunnan Agricultural University, China. The fungus was transferred to potato dextrose agar (PDA) medium and incubated at 28 °C in a temperature-controlled chamber for 7 days, and then stored in a refrigerator. The pectin, cellulose, and galacturonic acid used in this study were purchased from Tokyo Chemical Industry (Tokyo, Japan), Sigma Co. (St. Louis, MO, USA), and Ryon Co. (Shanghai, China), respectively.

### 4.2. Preparation of Spore Suspension

Spore suspensions of plant pathogens were obtained from 14-day-old cultures that were collected by adding 10 mL of sterile water to each Petri dish and rubbing the surface with a sterile L-shaped spreader. The suspension was filtered through four layers of cheesecloth.

### 4.3. Greenhouse Experiments

Faba bean seeds were soaked for 24 h at room temperature, germinated at 25 °C, and sown in sterile quartz sand soaked in Hoagland nutrient solution. Once the faba bean seedlings had grown 4–6 leaves, they were transplanted into 2 L containers containing various cinnamic acid concentrations. Treatments included 0 (control), 50, 100, and 200 mg·L^−1^ of cinnamic acid. There were three replications of these treatments, resulting in 36 plants (3 replicate pots × 3 seedlings × 4 concentrations). The controls received Hoagland nutrient solution instead of cinnamic acid treatment, and the experiments were conducted under 24 h pump ventilation. After 2 days, the FOF spore suspension (1 × 10^6^ cfu·mL^−1^) was added to the nutrient solution.

### 4.4. Determination of Faba Bean Growth Indices

The faba beans were sampled at the branching stage. The number of leaves per plant, maximum leaf length and width, height, main root length, shoot dry weight, and root dry weight were measured.

### 4.5. Measurement of the Incidence of Fusarium Wilt

There were three replicates for four treatments, resulting in 72 plants (3 replicate pots × 6 seedlings × 4 concentrations) at the branching stage. Following the survey, the incidence of fusarium wilt and the disease index were calculated. The degree of disease was divided into 5 levels: 0 corresponding to no infection; 1 corresponding to initial symptoms of fusarium wilt, with the base of the stem or root (except the main root) being slightly discolored; 2 corresponding to the base of the stem or the root having lesions, though not being contiguous; 3 corresponding to 1/3–1/2 of the stem base or root showing lesions, discoloration, or wilt, and lateral roots being significantly reduced; 4 corresponding to the base of the stem being surrounded by lesions or most of the roots being discolored and wilted; and 5 corresponding to plants having died or totally wilted. The incidence of fusarium wilt and the disease index were calculated, using the following formulas:

$$\mathrm{Incidence}=\frac{\mathrm{Number}\;\mathrm{of}\;\mathrm{diseased}\;\mathrm{plants}}{\mathrm{total}\;\mathrm{number}\;\mathrm{of}\;\mathrm{plants}\;\mathrm{investigate}}\;\times 100\%$$

$$\mathrm{Disease}\;\mathrm{index}=\frac{\Sigma \left(\mathrm{Number}\;\mathrm{of}\;\mathrm{diseased}\;\mathrm{plants}\;\mathrm{at}\;\mathrm{each}\;\mathrm{level}\times \;\mathrm{level}\right)}{2a\mathrm{The}\;\mathrm{highest}\;\mathrm{level}\;\times \mathrm{total}\;\mathrm{number}\;\mathrm{of}\;\mathrm{plants}\;\mathrm{investigated}}\;\times 100$$

### 4.6. Determination of Antioxidant Enzymes and Membrane Lipid Peroxidation

Fresh root samples were used to determine peroxidase (POD), catalase (CAT), and malondialdehyde (MDA) activities, according to the method of Li [51].

POD activity was measured by the guaiacol method. A 1.0 g sample was ground into a homogenate with an appropriate amount of phosphate buffer. The homogenate was centrifuged at 3000 rpm for 10 min and the supernatant was transferred to a 25 mL volumetric flask. We added 2.9 mL of 0.05 M phosphate buffer, 1.0 mL of 2% H_2_O_2_, 1.0 mL of 0.05 M guaiacol, and 0.1 mL of enzyme solution, and the solution was held in a 34 °C water bath for 3 min, and then was rapidly diluted once. The absorbance at 470 nm was recorded at 1 min intervals for 5 min.

CAT activity was assayed by potassium permanganate titration. A 1 g aliquot of the material and a small amount of phosphate buffer (pH 7.8) were ground into a homogenate, transferred to a 25 mL volumetric flask, and centrifuged at 4000 rpm for 15 min. A 2.5 mL aliquot of the supernatant was added to a measuring flask, and 2.5 mL of 0.1 M H_2_O_2_ was added. After 10 min of incubation in a constant-temperature water bath at 30 °C, 2.5 mL of 10% H_2_SO_4_ was immediately added. The solution was then titrated with a 0.1 M KMnO_4_ standard solution until a pink colour appeared. Enzyme activity was expressed in milligrams of H_2_O_2_ decomposed per gram of sample in 1 min.

A 0.5 g portion of a plant sample was added to 5 mL of 5% TCA, and the homogenate was centrifuged at 3000 rpm for 10 min after grinding. After mixing 2 mL of the supernatant and 2 mL of 0.67% TBA, the mixture was boiled in a water bath at 100 °C for 30 min, and then centrifuged once again after cooling. Absorbance values at 450, 532, and 600 nm were measured, after which the MDA content was calculated.

### 4.7. Determination of Pathogenesis-Related Protein activities

We measured chitinase activity using a chitinase kit purchased from Nanjing Jiancheng Bioengineering Institute, and 1 unit of chitinase activity was defined as the amount of chitin that decomposed per gram of tissue per hour to produce 1 mg of N-acetyl-d-(+)-glucosamine. β-1,3-glucanase activity was measured using a β-1,3-glucanase kit purchased from Beijing Soleil Technology Co., Ltd. (Beijing, China); 1 unit of β-1,3-glucan enzyme activity was defined as 1 mg of reducing sugar produced per gram of tissue per hour.

### 4.8. Extraction and Quantification of Mycotoxin

FOF was grown in Richard’s medium [52], containing 10 g of KNO_3_, 0.02 g of FeSO_4_, 5 g of KH_2_PO_4_, 2.5 g of MgSO_4_, 34 g of glucose, and 1000 mL of distilled water to produce mycotoxin. The strain was transferred to a PDA plate medium and cultured at 28 °C for 7 days. The diameter of the strain flake was 9 mm. The flakes were transferred to 250 mL Erlenmeyer flasks with 125 mL of culture medium, and eight plates were placed in a shaker for 15 days at 28 °C with rotations at 180 rpm. The culture medium was centrifuged at 5000 rpm for 10 min, and the supernatant was filtered using a 0.45 μm filter to remove mycelia and spores, after which the supernatant was collected and autoclaved to obtain the crude FOF toxin solution.

An equal volume of ethyl acetate was added to the crude toxin, shaken for 2 min, and precipitated for 30 min to estimate mycotoxin production. The organic phase was collected and centrifuged at 4000 rpm for 10 min. The supernatant was dried at 40 °C, and the entire dried product was dissolved in 5 mL of ethyl acetate. Absorbance was determined at a wavelength of 269 nm.

### 4.9. Measurement of Pathogenesis-Related Hydrolytic Enzyme Activities

The protease-producing culture medium contained 1% of inducing substrate (pectin and cellulose), 0.2 g of MgSO_4_·7H_2_O, 0.4 g of KH_2_PO_4_, 0.2 g of KCl, 1 g of NH_4_NO_3_, 0.01 g of FeSO_4_, 0.01 g of ZnSO_4_, and 0.01 g of MnSO_4_ in distilled water, being a total volume of 1 L [53]. We added 25 mL of the enzyme-producing medium to 100 mL Erlenmeyer flasks, and FOF flasks (diameter: 9 mm) were cultured for 7 days at 28 °C and 200 rpm. The culture medium was collected and centrifuged at 4000 rpm for 10 min, and the supernatant was filtered through a 0.45 μm filter. The filtrate was the crude enzyme solution, which was stored at 4 °C until measurements were conducted.

We prepared 3,5-dinitrosalicylic acid (DNS) reagents according to a previously described method [54]. We added 3.15 g of DNS to 500 mL of water while stirring for 5 s and heated the solution to 45 °C. 100 mL of 0.2 g·mL^−1^ sodium hydroxide solution was then gradually added while stirring until the solution was transparent. The solution temperature did not exceed 48 °C during the addition of the sodium hydroxide. We added 91.0 g of sodium nitrate potassium tartrate, 2.5 g of phenol, and 2.5 g of anhydrous sodium sulphite, and placed the solution in a water bath to heat it to 45 °C while adding 300 mL of water with constant stirring until the material was completely dissolved. Finally, the solution was cooled to room temperature and distilled water was added to a final volume of 1L. The solution was stored at room temperature in the dark for 7 days before use. A unit of enzyme activity was defined as 1 μmol of galacturonic acid produced by pectinase hydrolysis per min, or the amount of enzyme required to produce 1 μmol of glucose per min.

### 4.10. Statistical Analysis

All data were analyzed using Microsoft Excel 2010 software (Microsoft Corp., Redmond, WA, USA) and SPSS software (ver. 20.0; SPSS Inc., Chicago, IL, USA). The least significant difference test was used to determine differences between the treatments, at a significance level of *p* < 0.05.

## 5. Conclusions

Cinnamic acid significantly stimulated FOF, which produced fusaric acid and hydrolase, inhibited faba bean seedling growth, and stimulated membrane lipid peroxidation in faba beans’ roots, destroying the host defence system against the pathogen. This process may explain why, in this study, plant morbidity and disease indices were found to increase, compared with the control.

## Figures and Tables

**Table 1:** Effects of cinnamic acid on the growth of faba bean seedlings after being inoculated with *Fusarium oxysporum* f. *fabae* (FOF). Data are average values, with bars representing standard errors of three replicates. Different letters for each index indicate significant differences at a level of *p* < 0.05, according to Duncan’s test. There are no significant differences among values labeled “ab”, “a”, and “b”.

Growth Parameter	0 mg·L^−1^	50 mg·L^−1^	100 mg·L^−1^	200 mg·L^−1^
Leaf number per plant	21.67 ± 0.58a	16.33 ± 2.89b	13.00 ± 1.00c	11.83 ± 0.29d
Max leaf length (cm)	8.73 ± 0.38a	6.80 ± 0.61b	6.47 ± 0.38b	4.50 ± 0.10c
Max leaf width (cm)	5.43 ± 0.51a	4.13 ± 0.12b	3.77 ± 0.55b	2.93 ± 0.15c
Height (cm)	39.23 ± 0.49a	33.23 ± 1.48b	27.33 ± 1.11c	22.83 ± 0.58d
Main root length (cm)	18.33 ± 1.46a	17.53 ± 0.42a	16.77 ± 0.45b	13.23 ± 0.31c
Shoot dry weight (g)	2.23 ± 0.05a	1.66 ± 0.11b	0.92 ± 0.21c	0.71 ± 0.08c
Root dry weight (g)	0.54 ± 0.03a	0.39 ± 0.05b	0.20 ± 0.05c	0.14 ± 0.04c
Root–shoot ratio (%)	0.24 ± 0.02a	0.23 ± 0.02a	0.22 ± 0.01ab	0.20 ± 0.02b

**Table 2:** Effect of cinnamic acid on seedling root physiological activity and content of malondialdehyde (MDA) after being inoculated with FOF. Data are average values, with bars representing standard errors of three replicates. Different letters for each index indicate significant differences at *p* < 0.05, according to Duncan’s test. There is no significant difference among values labeled “ab”, “a”, and “b”.

Concentration (mg·L^−1^)	POD Activity (μ·g^−1^·min^−1^)	CAT Activity (mg·g^−1^·min^−1^)	MDA Content (μmol·g^−1^)
0	23.33 ± 0.58a	0.51 ± 0.02a	12.77 ± 1.54d
50	19.33 ± 0.58b	0.43 ± 0.04b	15.89 ± 0.92c
100	16.33 ± 1.53c	0.28 ± 0.02c	17.94 ± 0.90b
200	12 ± 1.73d	0.19 ± 0.03d	21.04 ± 0.47a
